# Atrophic Patterns of the Frontal-Subcortical Circuits in Patients with Mild Cognitive Impairment and Alzheimer’s Disease

**DOI:** 10.1371/journal.pone.0130017

**Published:** 2015-06-11

**Authors:** Hui Zhao, Xiaoxi Li, Wenbo Wu, Zheng Li, Lai Qian, ShanShan Li, Bing Zhang, Yun Xu

**Affiliations:** 1 Department of Neurology and Radiology, Affiliated Drum Tower Hospital, Nanjing University Medical School, Nanjing, Jiangsu, PR China; 2 The State Key Laboratory of Pharmaceutical Biotechnology, Nanjing University, Nanjing, Jiangsu, PR China; 3 Jiangsu Key Laboratory for Molecular Medicine, Nanjing University Medical School, Nanjing, China; 4 Nanjing clinic medicine center for neurological and psychiatric diseases, Nanjing, China; 5 Department of Neurology and Radiology, Affiliated Drum Tower Hospital, Nanjing Medical University, Nanjing, Jiangsu, PR China; Nathan Kline Institute and New York University School of Medicine, UNITED STATES

## Abstract

Atrophy of the cortical thickness and gray matter volume are regarded as sensitive markers for the early clinical diagnosis of Alzheimer’s disease (AD). This study aimed to investigate differences in atrophy patterns in the frontal-subcortical circuits between MCI and AD, assess whether these differences were essential for the pathologic basis of cognitive impairment. A total of 131 individuals were recruited, including 45 with cognitively normal controls (CN), 46 with MCI, and 40 with AD. FreeSurfer software was used to perform volumetric measurements of the frontal-subcortical circuits from 3.0T magnetic resonance (MR) scans. Data revealed that both MCI and AD subjects had a thinner cortex in the left caudal middle frontal gyrus and the left lateral orbitofrontal gyrus compared with CN individuals. The left lateral orbitofrontal gyrus was also thinner in AD compared with MCI patients. There were no statistically significant differences in the cortical mean curvature among the three groups. Both MCI and AD subjects exhibited smaller bilateral hippocampus volumes compared with CN individuals. The volumes of the bilateral hippocampus and the right putamen were also smaller in AD compared with MCI patients. Logistic regression analyses revealed that the left lateral orbitofrontal gyrus and bilateral hippocampus were risk factors for cognitive impairment. These current results suggest that atrophy was heterogeneous in subregions of the frontal-subcortical circuits in MCI and AD patients. Among these subregions, the reduced thickness of the left lateral orbitofrontal and the smaller volume of the bilateral hippocampus seemed to be markers for predicting cognitive impairment.

## Introduction

Alzheimer’s disease (AD) is one of the most common neurodegenerative disorders in the elderly. The pathological features of AD are amyloid deposition and neurofibrillary tangles (NFTs) in the brain, which lead to progressive neuronal damage and subsequent cerebral atrophy in the brain [1]. Individuals with mild cognitive impairment (MCI) have impaired memory and cognitive profiles compared with their contemporaries, but do not yet fulfill the criteria of AD [2]. Individuals with memory impairment and MCI seem to be more vulnerable to progression toward AD. Specifically, the annual conversion is 15–20%, compared with 1–2% in healthy elderly individuals [3]. Some neuropathological studies even suggested that amnestic MCI might be a transitional state between healthy aging and AD [4]. Therefore, studies started to focus on identifying markers for the progression of MCI to AD[5–7].

Structural magnetic resonance imaging (MRI) studies have proved useful for evaluating the prognosis of individuals with MCI and AD. Previous studies found that grey matter (GM) and cortex atrophy in brain regions such as the hippocampus, entorhinal cortex, and medial temporal cortex was observed in most patients diagnosed with MCI and early-stage AD [8,9]. Some subcortical structures, such as the amygdala, putamen, caudate, and thalamus, are also affected in individuals with MCI and AD [10–12].

The frontal-subcortical circuits include the frontal cortex gyrus, cingulate gyrus, thalamus, caudate, putamen, pallidum, hippocampus, amygdale, and accumbens nucleus [13–14], which function as making and planning complex behavioral strategies, making decisions, and neuropsychiatric manifestations. Recent research in the 5×FAD (five familial AD mutations) mouse model of AD found that early cognitive deficits related to the frontal cortex appeared before hippocampal-dependent learning and memory impairment, and were consistent with the neuropathological processes associated with AD [15]. However, few studies have assessed the changes that occur in subregions in the frontal-subcortical structure in patients with MCI and AD.

The current study used the FreeSurfer automated software package to analyze frontal-subcortical structural MRI data obtained from cognitively normal control, MCI and AD patients. The goal was to characterize the morphology of the frontal-subcortical structure in patients with MCI and AD. We hypothesized that the pattern of atrophy in some subregion was heterogeneous in MCI and AD, and that this atrophy pattern correlated with poorer cognitive test results. These observations might be helpful for physicians to predict cognitive deficits and early after the initiation of MCI.

## Subjects and Methods

### Patient enrollment

Between October 2010 and February 2014, 40 AD patients, 46 MCI patients, and 45 cognitively normal controls (CN) were enrolled in this study. All 131 subjects were recruited from the Department of Neurology of the Affiliated Drum Tower Hospital of Nanjing University Medical School. The ethics committees of the Affiliated Drum Tower Hospital of Nanjing University Medical School approved the study protocol (clinical trials government identifier: NCT01364246). All patients provided written informed consent.

All subjects were examined using a standardized protocol, including a neuropsychological screening, a whole brain MRI, a general medical, and a neurological examination that was performed by a neurologist. The clinical diagnosis of probable AD was made by a multidisciplinary consensus meeting according to the National Institute of Neurological and Communicative Disorders and Stroke and the Alzheimer’s Disease and Related Disorders Association (NINCDS-ADRDA) criteria [16]. Patients with dementia had impaired memory and a clinical dementia rating scale(CDR) score of ≥1.0.

MCI was diagnosed according to the revised Petersen’s criteria for MCI [2]. The criteria were as follows: subjective memory complaints, a clinical dementia rating scale (CDR) score of 0.5, and impaired memory function in memory tests (1.5 standard deviation [SD] points below the mean of age- and education-adjusted norms). Individuals with any cerebrovascular abnormalities, as determined by T2WI, or a history of brain injury or alcoholism were excluded from the study. Subjects with visible white matter hyperintensity (WMH; grade 2 or more according to a reported rating scale [17]) were also excluded. All of the normal control subjects had no cognitive complaints, and no neurological or psychiatric disorders. All participants were right-handed.

### Neuropsychological assessments

The cognitive function of all subjects was evaluated using a standardized neuropsychological test battery, including the mini mental state examination (MMSE), Montreal cognitive assessment (MOCA), CDR, neuropsychiatric inventory (NPI), activities of daily living scale (ADL), Hamilton depression rating scale (HAMD), and digit-symbol coding from the Wechsler adult intelligence scale (WAIS).

### MRI acquisition

MRI examinations were performed at 3 Tesla using an eight-channel phased array coil (Achieva 3.0T TX dual-source parallel RF excitation and transmission technology, Philips Medical Systems, The Netherlands). Three-dimensional high-resolution sagittal T1W with turbo fast echo (3D-T1TFE) acquisition was performed with repetition time (TR), echo time (TE), and inversion time (TI) of 9.8, 4.6, and 900 msec, respectively, a flip angle of 8°, 1.0 mm isotropic resolution, and 192 slices. The total scan time was 6 min and 43.6 sec.

### Image preprocessing

The cortical thickness and subcortical structure volume were estimated using FreeSurfer v5.1.0 (http://www.nmr.mgh.harvard.edu/freesurfer/). The overall process and analysis pipeline have been described elsewhere (http://surfer.nmr.mgh.harvard.edu). First, the high-resolution 3D T_1_TFE volumes were converted to FreeSurfer format (mgh file), normalized for intensity and resampled to isotropic voxels of 1mm^3^. Next, the skull was removed using a skull-stripping algorithm and segmented into three tissue types (white matter, grey matter and CSF). Third, cortical thickness measurements were obtained by reconstructing representations of the grey/white matter interface and the cortical surface and then by calculating the distance between these surfaces at each point across the curtail mantle. For this cortical thickness measurements purpose, a warping of subject cortical surfaces to a reference surface with known localization of cortical structures is required. An FS standard averaged cortical surface template (FSaverage template) and nonlinear procedure were used[18]. In order to obtain difference maps of cortical thickness, the data were smoothed on the level of the sphere using Gaussian smoothing kernel with a full-width half maximum of 10mm.

35 subcortical volumes (“aseg.stats” files) as well as cortical thickness values of 68 structures (“aparc.stats” files) based on Desikan-Killiany atlas were extracted from FreeSurfer[19] for further statistical analysis. Both the cortical[20] and the subcortical [21] labeling use the same basic algorithm. The final segmentation is based on both a subject-independent probabilistic atlas and subject-specific measured values. The atlas is built from a training set, i.e., a set of subjects whose brains (surfaces or volumes) have been labeled by hand. These labels are then mapped into a common space (Talairach space for volumes and spherical space for surfaces) to achieve point-to-point correspondence for all subjects. Note that a "point" is a voxel in the volume or a vertex on the surface. At each point in space, there exists the label that was assigned to each subject and the measured value (or values) for each subject.

### Statistical analyses

**Fig 1:**
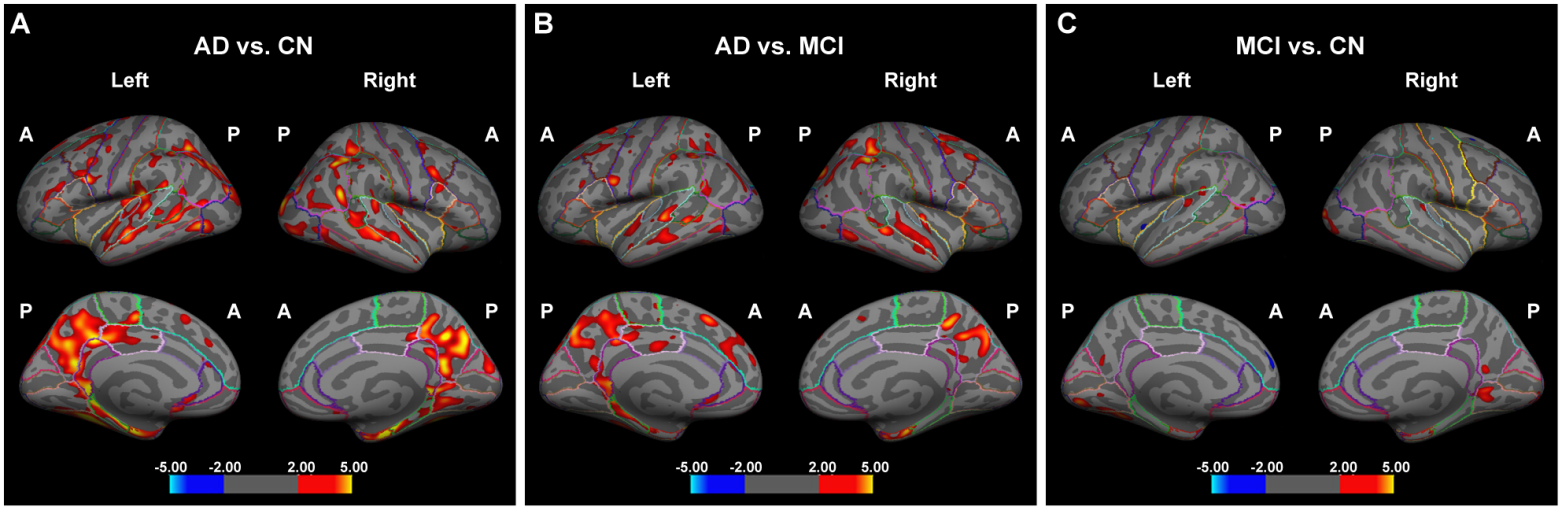
Analysis of cortical thickness to differentiate between AD patients and cognitively normal (CN) controls (A), AD patients and MCI patients (B), and MCI patients and CN controls (C) using FreeSurfer. A decreasing cortical thickness is indicated by red and yellow, and an increased cortical thickness is shown in cyan and blue. A, anterior; P, posterior.

The group differences of cortical thickness were explored with two types of analysis: a vertex-based analysis and a ROI-based one. The whole brain vertex-wise analysis is a point-by-point group comparison of thickness across the cortical surface, without any priori hypothesis, starting with the average images of each group. The statistical analysis was performed using Qdec, a module of Freesurfer developed to design and execute surface analysis. The design matrix used in Qdec had two regressors for each comparison. Then each of the regressor is the independent variable, the significant atrophy between groups is the dependent variable also known as a "measured variable" or "outcome variable". We had three groups, so three mtx files were generated, including NC-AD (1 0–1), NC-MCI (1–1 0) and MCI-AD (0 1–1), corresponding to each comparison. Because there were no significantly differences in ages among three groups, so we had not introduced it as a nuisance factor in statistical analysis. To correct the multiple comparison, we performed Monte-Carlo cluster-based simulation with 5000 permutations comprising the synthesis of white Gaussian nosied on the estimated surface, smoothing and clustering to correct for multiple comparisons using a cluster threshold of p<0.01. Areas showing significant cortical thinning were superimposed on the template (flattened FSaverage pial surface); the color bar scale represents t values (Fig 1).

For each ROI, the mean cortical thickness in millimeters, the mean curvature in mm^-1^, the subcortical structure volume in cubic centimeters was extracted for subsequent statistical analysis. For the purposes of this study we focused on ROIs in the frontal subcortical circuit (14 in the frontal lobe, 8 in the cingulate, and 14 in the subcortical regions bilaterally). All statistical calculations were performed using SPSS 17.0 (Bizinsight, Shanghai, China). Data are presented as means ± SEM. To analyze the cortical thickness, the mean curvature and subcortical structure volume, intergroup (CN, MCI, and AD) differences were analyzed using one-way analysis of variance (ANOVA) followed by Student-Newman-Keuls tests. FDR correction for multiple testing was applied for determining significant differences among three groups,. Correlations between cognitive scores in MMSE tests and cortical thickness and subcortical structure volume were assessed by Pearson’s correlation coefficients(*r*). Multivariate logistic regression analysis was used to assess the associations between frontal-subcortical structure and AD. The data were expressed as odds ratios (OR) and 95% confidence intervals (CI) and probability values. Age, gender and ICV were taken in as co-variates in the statistical analysis. Statistical significance was accepted when *P* < 0.05.

## Results

### Neuropsychological assessment

As shown in Table 1, there were three groups in this study: 40 AD patients, 46 MCI patients, and 45 cognitively normal control individuals. There were no significant differences in the male-to-female ratio, age, education and estimated intracranial volume among groups (*P* > 0.05).

**Table 1:** Clinical characteristics and neuropsychological assessment of the study subjects (means ± SEM).

	CN (*n* = 45)	MCI (*n* = 46)	AD (*n* = 40)
Age	70.92 ± 9.49	73.22 ± 9.82	72.34 ± 9.13
Gender (male %)	26 (49.1%)	22 (47.8%)	14 (48.3%)
Education	12.01 ± 3.17	11.85 ± 3.17	11.57 ± 3.15
ICV(cm3)	1523 ± 87	1529 ± 126	1507 ± 120
MMSE	28.85 ± 1.11	24.63 ± 1.70*	20.41 ± 2.94* #
MOCA	27.00 ± 1.35	21.70 ± 1.75*	15.62 ± 3.58* #
ADL	21.30 ± 1.51	22.30 ± 2.04	31.93 ± 10.82* #
Hachinski	1.74 ± 1.25	4.15 ± 1.71	3.45 ± 2.22
HAMD	1.38 ± 2.30	2.87 ± 2.17	3.28 ± 3.54
CDR	3.28 ± 3.83	13.35 ± 5.86*	22.14 ± 11.51* #
Digit-symbol coding tasks	39.96 ± 8.52	24.87 ± 6.28*	17.48 ± 5.65* #
Digit span (in order)	8.04 ± 1.53	7.20 ± 1.39*	6.24 ± 2.16* #
Digit span (backward)	5.32 ± 1.31	4.09 ± 1.24*	3.45 ± 1.05* #
NPI	0.00 ± 0.00	0.15 ± 0.42	0.31 ± 0.54*

Values are presented as mean ± SD unless otherwise stated; CN, cognitively normal; MCI, mild cognitive impairment;

AD, Alzheimer’s dementia; MMSE, mini-mental state examination;

MOCA, Montreal cognitive assessment; ADL, activities of daily living scale; HAMD, Hamilton depression rating scale;

CDR, clinical dementia rating scale; NPI, neuropsychiatric inventory.

* *P* < 0.05 (uncorrected) vs. CN;

^#^ *P* < 0.05 (uncorrected) vs. MCI.

Patients with MCI and AD had significant lower MMSE, MOCA, digit-symbol coding tasks, digit span (in order), and digit span (backward) scores than did subjects in the CN group (*P* < 0.05, uncorrected). These scores were also lower in the AD group compared with MCI (*P* < 0.05, uncorrected). In contrast, patients with AD scored higher in the ADL assessments compared with MCI and CN group (*P* < 0.05, uncorrected). The MCI and AD group had higher CDR, HAMD, Hachinski and NPI scores than the CN group (*P* < 0.05, uncorrected), but only the CDR scores in AD group was higher than MCI group (*P* < 0.05, uncorrected),there was no significant difference in other scores between the MCI and AD groups (*P* > 0.05).

### Analysis of the frontal cortex

The bilateral frontal cortex and cingulate cortex were divided into various regions according to the Desikan-Killiany atlas. The differentiation between CN, MCI, and AD patients is shown in Fig 1.

The mean cortical thickness of all subregions in the frontal cortex is shown in Table 2 separately for CN, MCI, and AD patients. The frontal cortex was divided into 14 subregions (seven per hemisphere), and analysis revealed that the cortical thickness in the left caudal middle frontal gyrus and left lateral orbitofrontal gyrus was thinner in MCI and AD groups compared with CN (*P* < 0.05, FDR corrected). In addition, the cortical thickness of the left lateral orbitofrontal gyrus was thinner in the AD group compared with MCI (*P* < 0.05, FDR corrected). There was no significant difference in the cortical thickness in the other subregions among the three groups (*P* > 0.05). The cortical mean curvature for all subregions of the frontal cortex is shown separately in Table 3 for the CN, MCI, and AD patients. There was no significant difference among the groups (*P* > 0.05).

**Table 2:** Thicknesses of the frontal cortex in different groups (mean ± S.D, mm)

	CN (*n* = 45)	MCI (*n* = 46)	AD (*n* = 40)	Pearson-corr
L caudal middle frontal gyrus	2.94 ± 0.18	2.46 ± 0.13*	2.11 ± 0.14*	0.121^▲^
L lateral orbitofrontal gyrus	2.28 ± 0.20	1.83 ± 0.20*	1.29 ± 0.23* #	0.204^▲^
L medial orbitofrontal gyrus	2.15 ± 0.18	2.10 ± 0.23	2.08 ± 0.20	0.193^▲^
L parsorbitalis gyrus	2.16 ± 0.23	2.11 ± 0.21	2.08 ± 0.24	0.126
L rostral middle frontal gyrus	1.85 ± 0.16	1.62 ± 0.13	1.49 ± 0.13	0.220^▲^
L superior frontal gyrus	2.22 ± 0.17	2.20 ± 0.14	2.14 ± 0.17	0.242
L frontal pole gyrus	2.17 ± 0.25	2.20 ± 0.26	2.23 ± 0.28	-0.083
R caudal middle frontal gyrus	2.07 ± 0.14	2.02 ± 0.32	1.94 ± 0.16	0.186
R lateral orbitofrontal gyrus	2.19 ± 0.16	2.11 ± 0.33	2.11 ± 0.16	0.150
R medial orbito frontal gyrus	2.12 ± 0.20	2.05 ± 0.35	2.12 ± 0.17	0.044
R parsorbitalis gyrus	2.09 ± 0.20	2.02 ± 0.34	2.07 ± 0.24	0.066
R rostral middle frontal gyrus	1.85 ± 0.17	1.80 ± 0.28	1.84 ± 0.20	0.064
R superior frontal gyrus	2.20 ± 0.16	2.14 ± 0.34	2.12 ± 0.15	0.167
R frontal pole gyrus	2.14 ± 0.27	2.10 ± 0.35	2.17 ± 0.27	-0.019

The correlations between cortical thickness and MMSE used by Pearson’s correlation coefficient).

CN, cognitively normal; MCI, mild cognitive impairment; AD, Alzheimer’s dementia;

MMSE, mini-mental state examination; L, left; R, right.

* *P* < 0.05 (FDR corrected) vs. CN;

^#^ *P* < 0.05(FDR corrected) vs. MCI;

^▲^ *P* < 0.05.

**Table 3:** Mean curvature of the frontal cortex in different groups (means ± S.D,1/ mm).

	CN (*n* = 45)	MCI (*n* = 46)	AD (*n* = 40)	Pearson-corr
L caudal middle frontal gyrus	0.13 ± 0.01	0.18 ± 0.29	0.19 ± 0.27	-0.033
L lateral orbitofrontal gyrus	0.16 ± 0.01	0.21 ± 0.32	0.24 ± 0.37	-0.027
L medial orbitofrontal gyrus	0.17 ± 0.02	0.21 ± 0.23	0.24 ± 0.20	-0.032
L parsorbitalis gyrus	0.19 ± 0.03	0.23 ± 0.11	0.26 ± 0.14	-0.019
L rostral middle frontal gyrus	0.18 ± 0.02	0.28 ± 0.43	0.24 ± 0.29	-0.039
L superior frontal gyrus	0.15 ± 0.07	0.19 ± 0.19	0.22 ± 0.17	-0.038
L frontal pole gyrus	0.24 ± 0.05	0.21 ± 0.16	0.32 ± 0.23	-0.068
R caudal middle frontal gyrus	0.13 ± 0.01	0.21 ± 0.38	0.20 ± 0.33	-0.041
R lateral orbitofrontal gyrus	0.16 ± 0.01	0.24 ± 0.34	0.23 ± 0.31	-0.038
R medial orbito frontal gyrus	0.17 ± 0.20	0.24 ± 0.25	0.25 ± 0.17	-0.045
R parsorbitalis gyrus	0.18 ± 0.02	0.28 ± 0.43	0.24 ± 0.29	-0.038
R rostral middle frontal gyrus	0.17 ± 0.01	0.25 ± 0.24	0.23 ± 0.12	-0.052
R superior frontal gyrus	0.15 ± 0.01	0.24 ± 0.34	0.22 ± 0.15	-0.39
R frontal pole gyrus	0.24 ± 0.07	0.23 ± 0.25	0.32 ± 0.22	-0.066

The correlation between cortical mean curvature and MMSE was analysised by Pearson’s correlation coefficient.

CN, cognitively normal; MCI, mild cognitive impairment; AD, Alzheimer’s dementia;

MMSE, mini-mental state examination; L, left; R, right.

The correlations between the cognitive scores in the MMSE and the cortical thickness in subregions of the frontal cortex are shown in Table 2. The results indicated that there was a positive correlation between the MMSE scores and the cortical thickness in the left caudal middle frontal gyrus, the left lateral orbitofrontal gyrus, the left medial orbitofrontal gyrus and the left rostral middle frontal gyrus, (*P* < 0.05). In addition, there was a negative correlation between the MMSE scores and the mean cortical curve of the right caudal middle frontal gyrus (*P* < 0.05). There was no correlation between the MMSE score and other subregions (*P* > 0.05; Table 3).

The cingulate cortex was divided into eight subregions (four per hemisphere). The mean thickness and the mean cortical curve for all the subregions in the CN, MCI, and AD groups was shown in Table 4 and Table 5. There was no significance difference in all subregions among the three groups (*P* > 0.05).

**Table 4:** Cortical thicknesses of the cingulate cortex in different groups (means ± S.D, mm).

	CN (*n* = 45)	MCI (*n* = 46)	AD (*n* = 40)	Pearson-corr
L caudal anterior cingulate gyrus	2.29 ± 0.31	2.22 ± 0.39	2.14 ± 0.27	0.221
L isthmus cingulate gyrus	2.15 ± 0.18	2.09 ± 0.17	2.00 ± 0.25*	0.281▲
L posterior cingulate gyrus	2.25 ± 0.21	2.21 ± 0.17	2.10 ± 0.24	0.188
L rostral anterior cingulate gyrus	2.42 ± 0.20	2.46 ± 0.32	2.17 ± 0.30*	0.215▲
R caudal anterior cingulate gyrus	2.22 ± 0.30	2.09 ± 0.35	2.20 ± 0.32	0.086
R isthmus cingulate gyrus	2.04 ± 0.15	1.96 ± 0.32	1.94 ± 0.19	0.172
R posterior cingulate gyrus	2.14 ± 0.20	2.03 ± 0.36	2.06 ± 0.20	0.203
R rostral anterior cingulate gyrus	2.48 ± 0.34	2.40 ± 0.42	2.34 ± 0.33	0.165

The correlations between cortical thickness and MMSE were analysised by Pearson’s correlation coefficient.

CN, cognitively normal; MCI, mild cognitive impairment; AD, Alzheimer’s dementia;

MMSE, mini-mental state examination; L, left; R, right.

* *P* < 0.05 (FDR corrected) vs. CN;

^▲^P < 0.05.

**Table 5:** Mean curvature of the cingulate cortex in different groups (means ± S.D, 1/mm).

	CN (*n* = 45)	MCI (*n* = 46)	AD (*n* = 40)	Pearson-corr
L caudal anterior cingulate gyrus	0.15 ± 0.01	0.19 ± 0.33	0.15 ± 0.01	0.037
L isthmus cingulate gyrus	0.15 ± 0.01	0.20 ± 0.33	0.16 ± 0.02	0.022
L posterior cingulate gyrus	0.26 ± 0.02	0.21 ± 0.34	0.16 ± 0.01	0.027
L rostral anterior cingulate gyrus	0.16 ± 0.03	0.21 ± 0.22	0.15 ± 0.02	0.047
R caudal anterior cingulate gyrus	0.15 ± 0.01	0.24 ± 0.41	0.22 ± 0.37	-0.075
R isthmus cingulate gyrus	0.15 ± 0.01	0.23 ± 0.36	0.22 ± 0.31	-0.083
R posterior cingulate gyrus	0.16 ± 0.02	0.24 ± 0.36	0.22 ± 0.03	-0.071
R rostral anterior cingulate gyrus	0.15 ± 0.04	0.26 ± 0.51	0.23 ± 0.39	-0.069

The correlations between cortical mean curvature and MMSE were analysised by Pearson’s correlation coefficient.

CN, cognitively normal; MCI, mild cognitive impairment; AD, Alzheimer’s dementia;

MMSE, mini-mental state examination; L, left; R, right.

Table 4 indicated that there was a positive correlation between the MMSE scores and the cortical thickness in the left isthmus cingulate gyrus and left rostral anterior cingulate gyrus (*P* < 0.05). There was no relationship between the cortical thickness in other subregions in the cingulate cortex and MMSE scores (*P* > 0.05). Moreover, there was no correlation between the MMSE scores and the mean cortical curve for all subregions in the cingulate cortex (*P* > 0.05; Table 5).

The effects of cortical thickness in the different subregions of the frontal cortex on predicting cognitive impairment was assessed using the means of logistic regression analysis in all subjects. As shown in Table 6, the left lateral orbitofrontal gyrus (OR = 2.021, 95%CI = 1.019–4.979, *P* < 0.05) was a risk factor of cognitive impairment.

**Table 6:** Logistic regression analyses on association between the thickness of the frontal cortex and cognitive impairment.

	*P*	OR	95%CI
L caudal middle frontal gyrus	0.397	0.139	0.001–13.408
L lateral orbitofrontal gyrus	0.048*	2.021	1.019–4.979
L medial orbitofrontal gyrus	0.685	0.559	0.034–9.284
L parsorbitalis gyrus	0.933	0.885	0.050–15.722
L rostral middle frontal gyrus	0.149	20.104	0.150–4879.650
L superior frontal gyrus	0.112	85.074	0.352–20533.321
L frontal pole gyrus	0.128	4.516	0.649–31.416
R caudal middle frontal gyrus	0.217	0.057	0.001–5.384
R lateral orbitofrontal gyrus	0.177	0.045	0.001–1.052
R medial orbito frontal gyrus	0.713	1.799	0.079–40.889
R parsorbitalis gyrus	0.613	2.155	0.110–42.199
R rostral middle frontal gyrus	0.208	28.322	0.155–5184.830
R superior frontal gyrus	0.782	0.490	0.003–77.442
R frontal pole gyrus	0.347	2.844	0.322–25.102
Constant	0.008	6812.497	-

L, left; R, right; OR, odds ratio; 95%CI, 95% confidence interval;

*P < 0.05

### Analysis of the subcortical structures

The QDEC toolbox in FreeSurfer was used to analyze the surface of 14 subcortical structures (seven per hemisphere) in the CN, MCI, and AD groups. The mean volumes of all the subcortical structures in cubic centimeters were shown separately in Table 7 for the CN, MCI, and AD groups. Compared with the CN group, the volume of the right putamen and bilateral hippocampus was smaller in AD patients (*P* < 0.05, FDR corrected). The volume of the bilateral hippocampus and the right putamen was also smaller in the AD compared with MCI group (*P* < 0.05, FDR corrected). There was no significant difference in other subcortical structures (thalamus, caudate nucleus, amygdale, pallidum and accumbens) among the CN, MCI, and AD groups (*P* > 0.05).

**Table 7:** Volumes of the subcortical structures in different groups (mean ± S.D, mm^3^).

	CN (*n* = 45)	MCI (*n* = 46)	AD (*n* = 40)	Pearson-corr
L thalamus	5515.48 ± 763.86	5607.30 ± 995.85	6147.37 ± 3828.30	-0.084
L caudate	3330.22 ± 571.54	3488.21 ± 719.08	3184.00 ± 659.21	0.086
L putamen	5390.43 ± 798.80	5341.19 ± 872.82	5003.10 ± 1006.52	0.216
L pallidum	1409.17 ± 245.55	1330.55 ± 252.76	1329.03 ± 300.81	0.148
L hippocampus	2842.42 ± 1072.02	2438.77 ± 870.94*	2104.75 ± 971.83* #	0.188▲
L amygdala	1339.29 ± 430.64	1335.22 ± 485.75	1137.02 ± 395.99	0.152
L accumbens	523.13 ± 135.11	476.39 ± 152.49	429.28 ± 144.21	0.241
R thalamus	5423.96 ± 669.79	5481.80 ± 1028.01	5581.59 ± 1716.71	-0.029
R caudate	3496.51 ± 558.01	3594.37 ± 661.24	3297.59 ± 620.04	0.143
R putamen	5254.02 ± 949.42	5275.88 ± 995.04	4675.52 ± 1394.12* #	0.187▲
R pallidum	1409.72 ± 263.69	1366.11 ± 252.26	1237.59 ± 284.73	0.217
R hippocampus	2982.30 ± 1197.73	2552.91 ± 832.88*	2163.66 ± 1004.61* #	0.179▲
R amygdala	1584.07 ± 490.56	1588.95 ± 453.73	1368.87 ± 421.37	0.144
R accumbens	520.50 ± 136.42	488.40 ± 147.40	446.67 ± 164.17	0.193

The correlations between volumes and MMSE were analysised by Pearson’s correlation coefficient.

CN, cognitively normal; MCI, mild cognitive impairment; AD, Alzheimer’s dementia;

MMSE, mini-mental state examination; L, left; R, right.

*P < 0.05(FDR corrected) vs. CN;

^#^ *P* < 0.05(FDR corrected) vs. MCI;

^▲^ *P* < 0.05.

The correlations between the MMSE cognitive scores and the volume of the subcortical structures are shown in Table 7. The volume of the bilateral hippocampus,and right putamen was positively correlated with MMSE scores (*P* < 0.05, Table 7). Multivariate logistic regression analysis showed that the volume of the left and right hippocampus were risk factors for cognitive impairment (OR = 3.021, 95%CI = 1.001–7.005, *P* < 0.05; OR = 2.390, 95%CI = 1.990–5.995, *P* < 0.05; Table 8). Specifically, individuals with smaller hippocampal volumes had a higher possibility of lower cognitive test scores.

**Table 8:** Logistic regression analyses on the association between the subcortical structure volume and cognitive impairment.

	*P*	OR	95%CI
L thalamus	0.308	1.000	1.000–1.001
L caudate	0.304	1.001	0.999–1.001
L putamen	0.737	1.000	0.999–1.001
L pallidum	0.305	0.999	0.996–1.001
L hippocampus	0.047*	3.021	1.001–7.005
L amygdala	0.507	1.001	0.999–1.002
L accumbens area	0.302	0.997	0.992–1.002
R thalamus	0.814	1.000	0.999–1.001
R caudate	0.689	1.000	0.998–1.001
R putamen	0.300	1.000	1.000–1.001
R pallidum	0.257	0.999	0.996–1.001
R hippocampus	0.032*	2.390	1.990–5.995
R amygdala	0.591	1.000	0.998–1.001
R accumbens area	0.331	0.997	0.992–1.002
Constant	0.226	6.996	-

L, left; R, right; OR, odds ratio; 95%CI, 95% confidence interval;

*P < 0.05

## Discussion

This study provided a thorough understanding of the morphology of the frontal-subcortical region in patients with MCI and AD. We first used FreeSurfer software to identify that the atrophic patterns in the frontal-subcortical circuits were heterogeneous. Second, these atrophic patterns were correlated with impaired cognitive function. Finally, atrophy of the left lateral orbitofrontal gyrus was related to cognitive impairment. To the best of our knowledge, this is the first study to assess the patterns of atrophy in subregions of the frontal-subcortical circuits of AD patients.

Some factors affect cortical thickness, such as ageing and handedness [22–23]. Therefore, in the current study all the enrolled subjects were right-handed and there were no differences among the three groups regarding age, gender, and education.

Previous studies using the ROI-based MRI volumetric methods identified atrophy in the frontal lobe of AD patients [24]. However, changes in subregions of the frontal lobe of AD patients were unclear until now. To obtain a more detailed pattern of atrophy in the frontal-subcortical structure, the frontal cortex and cingulate cortex were divided into 14 subregions (seven per hemisphere) and eight subregions (four per hemisphere) respectively. The cortical thickness and mean curve of each subregion were also analyzed. The results showed that there was a significantly reduced thickness in the left caudal middle frontal gyrus and left lateral orbitofrontal gyrus in AD patients compared with CN individuals. Compared with CN, the thickness of the left caudal middle frontal gyrus and left lateral orbitofrontal gyrus was significant thinner in the MCI group. Moreover, only the lateral orbitofrontal gyrus had a different thickness among the three groups. Previous researchers found that the spatial patterns of cortical thickness in gray matter regions of the frontal, temporal, and parietal lobes in MCI and Alzheimer dementia were more pronounced in the left hemisphere [25,26]. Our results were consistent with these.

Mean curvature was the average of the two principle curvatures (1/radius of an inscribed circle)[27]. The mean curvature was the average mean curvature in folded regions, suggested the changes of sulcal shape. Studies in MCI and Alzheimer's disease showed that significantly lower average mean curvature (greater sulcal widening) in the temporal lobe with disease progression from MCI to AD[28]. But in our study the cortical mean curvature of subregions in the frontal cortex and cingulate cortex was not different among the CN, MCI, and AD groups, which suggests that compared with the mean curvature, the thicknesses of some subregions in the frontal cortex and cingulate cortex were more sensitive to the decline of congnitive.

The functions associated with the orbitofrontal cortex cover a diversity of sensory-related behaviors with roles in taste, olfaction, decision-making, mood, and aggressiveness [29–34]. Some studies observed NFT pathology in the orbitofrontal cortex of brain donors with AD, and this pathology was been thought to be related to the non-memory-related behavioral changes observed in AD [35]. Recent study demonstrated that the volume reduction of orbitofrontal cortex in Alzheimer's disease, which also reflected the attenuation of brain connectivity[7]. Furthermore the orbitofrontal cortex combined with the rhinal cortex for associative and contextual information signals [36], suggesting that the orbitofrontal cortex is important for memory formation. The current analyses suggested that atrophy of the left orbitofrontal cortex began during MCI and concerned with with the memory impairment. Further analyses suggested that the change ofthickness in this subregion might help neurologists monitor the progression of cognitive impairment and allow patients to be treated early.

The cingulate cortex is a part of the limbic system, and this region was traditionally considered to play a key role in affection and motivation. Recent studies showed that hypoglycemia damaged the isthmus cingulate and impaired memory [37]. In addition, a structural MRI showed that compared with AD patients, MCI patients had thinner cortices in the anterior cingulate [38]. Studies of the olfactory regions in MCI patients found that the orbitofrontal cortex and anterior cingulate cortex were the important parts of the olfactory processing structures [39]. Our data suggested that the thicknesses of subregions in cingulate cortex had no difference among three groups. But there was a positive correlation between the MMSE scores and the cortical thickness in the left isthmus cingulate gyrus and left rostral anterior cingulate gyrus. Because these subregions are connected to the olfactory tract and olfactory dysfunction is an early clinical and pathological feature of AD, these regions might be the more sensitive to early AD-related changes. Certainly more research is needed for better comprehending the changes of these subregions in cognitive impairment.

From our observations, we found that decreased thicknesses of the left caudal middle frontal gyrus, the left lateral orbitofrontal gyrus were evident in MCI patients, together with reduced the volumes of bilateral hippocampus. Furthermore, these regional shape changes also were related to cognitive deterioration. Till now, little was known about the change of these subregions in frontal gyrus and in MCI and AD. To better understand the function of subregions in this area, further study should be started.

The frontal-subcortical structure includes the thalamus, caudate, putamen, pallidum, hippocampus, amygdale, and nucleus accumbens. The hippocampus is the major structure related to memory, and progressive alterations in the formation of the hippocampus during the course of AD is a well established finding, particularly in the early or even preclinical stages of the disease [40–41]. Recently studies in mild cognitive impairment and Alzheimer's disease found that t obvious atrophy in hippocampal CA1 and the basolateral complex of amygdale, especially in MCI patients who converted to AD over time [42–43]. In addition to the hippocampus, the basal nuclei and thalamus participate in regulating motor behavior, as well as emotional, motivational, associative, and cognitive abilities. Recent studies have increased the breadth of functions ascribed to this region. Neuroimaging studies suggested that the amygdala atrophy occurred in MCI and AD, the degree of the amygdala atrophy is comparable to that of the hippocampus in the earliest clinical stages of AD[44–45]. The research about late-onset (LOAD) Alzheimer's Disease patients showed that the significant reductions in the accumbens volumes [46]. MR images showed the strongly reduced volumes of putamen and thalamus in Alzheimer's disease and the degenerative processes might contribute to cognitive decline[47]. The volumes of thalamus and caudate in familial Alzheimer's disease reduced at a presymptomatic stage[48]. The accumbens nucleus is a key component of the limbic striatal loop, and is related to cognition, emotion, and emotional behaviors such as addiction, fear, and reward [49]. Studies of Huntington’s disease found that atrophy of the nucleus accumbens was apparent in the pre-manifest stage [50]. Until now, changes in the nucleus accumbens in AD were not well defined. The current results revealed that the volumes of the right putamen, and hippocampus were smaller in AD patients compared with MCI and CN. And the volumes of the right putamen and bilateral hippocampus connected with cognitive discorder. In addition, the reduced hippocampal volumes could predict the progression from MCI to AD, consistent with previous studies[51]. These results also brought more interesting problems. For the study of brain cognitive fuction is very complicated, there still many work need to do.

The main limitation of the present investigation is that the number of subjects was relatively small, which limits the analysis of transformation in the structure of the cortical subregions. Furthermore, the method used for semi-automatic volumetry has value for comparing atrophy patterns in the subcortical structure in groups of subjects, but it could not easily address subregional variation in the structure of interest or evaluate changes in the subregions quantitatively. Larger cohorts and a novel method are required to investigate whether each subregion of the cortical structure is asymmetrical and to assess the relationship between subregion shrinkage and cognitive decline. Another limitation is that this was a cross-sectional study, and so it was difficult to judge whether atrophy of the frontal cortex and other subcortical structures was a primary or secondary phenomenon to the hippocampal or entorhinal cortex. Therefore, further investigations are needed. Future studies should be longitudinal in design to evaluate the relationship between the decline in memory and atrophy of the frontal-subcortical structures.

In conclusion, the present study demonstrated that the pattern of atrophy in subregions of the frontal-subcortical circuits in MCI and AD patients was heterogeneous. This heterogeneous atrophy was associated with cognitive impairment. Among these subregions, the reduced thickness of the left lateral orbitofrontal and left rostral anterior cingulate seemed to predict the progression of cognitive impairment, suggesting that these structures might be important for detecting the subtle effects of prodromal AD or to assess the effectiveness of therapeutic intervention for AD.
